# Hyperspectral reflectance imaging for nondestructive evaluation of root rot in Korean ginseng (*Panax ginseng* Meyer)

**DOI:** 10.3389/fpls.2023.1109060

**Published:** 2023-02-01

**Authors:** Eunsoo Park, Yun-Soo Kim, Mohammad Akbar Faqeerzada, Moon S. Kim, Insuck Baek, Byoung-Kwan Cho

**Affiliations:** ^1^ Department of Biosystems Machinery Engineering, College of Agricultural and Life Science, Chungnam National University, Daejeon, Republic of Korea; ^2^ R&D Headquarters, Korea Ginseng Corporation, Yuseong, Daejeon, Republic of Korea; ^3^ Environmental Microbial and Food Safety Laboratory, Agricultural Research Service, United States Department of Agriculture, Beltsville, MD, United States; ^4^ Department of Smart Agricultural System, College of Agricultural and Life Science, Chungnam National University, Daejeon, Republic of Korea

**Keywords:** near-infrared hyperspectral imaging, non-destructive measurement, spectral analysis, plant phenomics, ginseng, root rot

## Abstract

Root rot of *Panax ginseng* caused by *Cylindrocarpon destructans*, a soil-borne fungus is typically diagnosed by frequently checking the ginseng plants or by evaluating soil pathogens in a farm, which is a time- and cost-intensive process. Because this disease causes huge economic losses to ginseng farmers, it is important to develop reliable and non-destructive techniques for early disease detection. In this study, we developed a non-destructive method for the early detection of root rot. For this, we used crop phenotyping and analyzed biochemical information collected using the HSI technique. Soil infected with root rot was divided into sterilized and infected groups and seeded with 1-year-old ginseng plants. HSI data were collected four times during weeks 7–10 after sowing. The spectral data were analyzed and the main wavelengths were extracted using partial least squares discriminant analysis. The average model accuracy was 84% in the visible/near-infrared region (29 main wavelengths) and 95% in the short-wave infrared (19 main wavelengths). These results indicated that root rot caused a decrease in nutrient absorption, leading to a decline in photosynthetic activity and the levels of carotenoids, starch, and sucrose. Wavelengths related to phenolic compounds can also be utilized for the early prediction of root rot. The technique presented in this study can be used for the early and timely detection of root rot in ginseng in a non-destructive manner.

## 1 Introduction

Ginseng is a medicinal herb whose main active components, ginsenosides, improve cardiac health, blood circulation, hypoxia, and stress. Processed ginseng is distributed in dried, steamed, and powdered forms (Li, 1995). In 2018, 86,223 tons of fresh ginseng were distributed worldwide, and the market size of ginseng-related products (which is increasing every year) was approximately 5,900 million USD (Baeg, 2022).

Ginseng is a slow-growing perennial herb with an optimal harvest age of 4–5 years for maximum marketability. However, the quality and quantity of ginseng have decreased by 30–60% owing to various soil-mediated root diseases (Young Sook et al., 2012; Farh et al., 2018). Root rot caused by *Cylindrocarpon destructans* is the main soil-mediated disease affecting ginseng crops in Korea, and repeated cultivation of ginseng leads to a very high possibility of infection (Jang et al., 2010).

*Cylindrocarpon destructans* is a soil-borne fungus that causes considerable damage to ginseng crops. The causative agent can overwinter in soil during the resting season, so that existing and replanted crops can be re-infected each season. A common symptom of *C. destructans* infection is a dark brown discolored area at the tip of the tap root that extends to the crown over time. Occasionally, this rot affects all parts of the root. As the infection progresses, the outer surface of the root is damaged and the inside completely decomposes, leaving a hollow root in the soil. In the final stages of the infection, the leaves turn yellow and wither, and the tip of the stem can be easily separated from the crown (Farh et al., 2018).

Soil-borne diseases (such as root rot) are difficult to control during crop cultivation. Therefore, the causative fungal species and its distribution in the soil are checked before planting, and its spread must be managed through biological and chemical control agents. However, the superficial symptoms of infection typically do not appear until harvest. As such, the general method of prevention involves periodically checking and controlling the density of the pathogens in the soil. This method is skill- and time-intensive and is very inefficient for frequently monitoring a large area of the field. Therefore, it is important to develop a technique that enables farmers to detect soil-borne fungal infection through changes in aboveground plant parts.

Generally, crop phenotyping involves the quantification of phenotypic changes using RGB images. During the early stages of infection, root rot is difficult to detect through aboveground changes based on general RGB imaging technology. Hyperspectral imaging (HSI) technology is an alternative to RGB imaging that can detect minute changes in infected ginseng plants. When implemented as an imaging technique using spectroscopic technology, HSI can be used to detect chemical changes in an object (Bock et al., 2010). Recently, HSI technology has been applied for crop phenotyping and remote sensing in the agricultural field, and it is being used to predict various abiotic and biotic stresses.

In the field of plant pathology, HSI systems are widely used to develop technologies that can detect fungal diseases at the scale of the tissue, leaf, single plant, and canopy (Thomas et al., 2018b). In particular, several recent studies have investigated disease detection at the plant and canopy scales. For example, one study investigating aboveground infection used a deep convolutional neural network (CNN) to identify the differences between soybean crops resistant to charcoal rot *(Macrophomina phaseolina*) (Nagasubramanian et al., 2019). Another study used generative adversarial nets to facilitate the early detection of tomato spotted wilt virus infection (Wang et al., 2019a). Drones have been used for the early detection of bacterial spot (*Xanthomonas perforans*) in tomato plants (Abdulridha et al., 2020a); early prediction of powdery mildew in barley at the canopy scale (Thomas et al., 2018a); detection of powdery mildew disease in squash; detection and prediction of the severity level (disease stage) of powdery mildew disease in wild pumpkin (Abdulridha et al., 2020b); and prediction of early blight (*Alternaria solani*) in potato plants in farmlands (van de Vijver et al., 2020). Researchers have developed the means to detect Fusarium head blight (FHB) in wheat, compare the resistance of wheat plants to FHB (Alisaac et al., 2018), and automatically detect yellow rust disease in wheat in farmlands (Zhang et al., 2019). Research has also been conducted on the detection and disease indexing of leaf spot disease in peanut (Chen et al., 2019) using HSI.

In a study on the prediction of root infection using aboveground data, the researchers utilized recursive feature elimination (RFE) based on the HSI of the visible/near-infrared (Vis/NIR) region (400–1000 nm) to facilitate the early detection of *Rhizoctonia solani* infection in sugar beets (Reynolds et al., 2012; Barreto et al., 2020). Infection was mainly predicted based on changes in the levels of carotenoids (513 nm), nitrogen (637, 700 nm), and soluble solids (584 nm); peroxidase activity (593 nm) in the roots; and peroxidase-related changes in chlorophyll (656 nm). Another study used the CNN model to detect clubroot infection in cabbage (Feng et al., 2022) and reported the possibility of predicting soil-borne infections in crops through the HSI of aboveground plant parts. In other cases for trees, multispectral imaging technique was used to observe *Phellinus weirii* (laminated root rot) in the forest (Leckie et al., 2004) and predict the white root rot of avocado trees (Pérez-Bueno et al., 2019), and hyperspectral imaging technique was applied to detect *Armillaria* genus (root rot) in vines (Calamita et al., 2021) and *Verticillium* wilt in olive trees (Calderón et al., 2013). Most of the current studies have focused on image-based detection for the obvious symptoms of biotic stress in plants. However, the comprehensive study on developing spectral imaging method for the detection of early symptom of plant biotic stress has not been investigated.

In this study, we developed a technique for the early detection of root rot in ginseng using HSI, which can sensitively identify biochemical changes in crops. We performed laboratory experiments to identify phenotypic factors (such as biochemical information) related to root rot in ginseng. Soil samples infected with root rot disease were collected from the field and divided into sterile and non-sterile groups. Following this, 1-year-old ginseng plants were transplanted into the soil samples and divided into two groups: normal and infected. The hyperspectral data of ginseng leaves were obtained periodically (through HSI in the 400–1800 nm range) and were used to investigate the possibility of predicting root rot infection in ginseng.

## 2 Materials and methods

### 2.1 Plant materials, environmental conditions, and experimental treatments

One-year-old ginseng seedlings (*Panax ginseng* variety ‘Chunpoong’) harvested in mid-March 2020 were provided by the R&D headquarters of the Korea Ginseng Corporation in Daejeon. Soil samples infected with root rot were collected from a ginseng garden in Goesan (Chungcheongbuk-do, Republic of Korea) and divided into a control group (120 °C, autoclaved for 20 min, 2 times) and an infected group (not autoclaved). The seedlings were planted in these soil samples. Each seedling was transplanted into a small pot and grown in a growth room chamber for 5 weeks (22 ± 2 °C, 60–70% humidity, 16:8 photoperiod with 15000 Lx light intensity). At the end of the growth period, a real-time PCR test was performed to confirm the active state of pathogens in the soil and ginseng tissue samples Lee S. H. (2021).

### 2.2 HSI system and image acquisition

In this study, two HSI systems were used in reflectance mode to collect hyperspectral images of ginseng plants in different ranges: Vis/NIR (400–1,000 nm) and short-wave infrared (SWIR; 1,000–2,500 nm). The other settings were the same as those used in a previous study (Park et al., 2021).

### 2.3 Data extraction

The Vis/NIR and SWIR images of 96 samples (control, 53; infected, 43) were obtained. HSI spectral data were extracted from the leaves of ginseng plants and averaged for each leaf. The control data consisted of 1272 (Vis/NIR) and 1326 (SWIR) images extracted from each leaf area of ginseng leaves in the control group. The data for the infected group included 949 (Vis/NIR) and 861 (SWIR) images. For calibration, we included 843 (879) and 628 (558) sample images in the Vis/NIR (SWIR) regions for the control and infected groups, respectively. For validation, we included 429 (447) and 321 (303) sample images in the Vis/NIR (SWIR) regions for the control and infected groups, respectively. MATLAB (Version R2019a, Mathworks, Natick, MA, USA) was used for data processing.

### 2.4 Multivariate analysis for classification

#### 2.4.1 Partial least squares discriminant analysis

Partial least squares regression (PLSR) is a type of regression analysis that identifies the model with the most significant correlation between latent variables (LVs) of the input (hyperspectral data) and output data (reference values for ginseng plants based on exposure to control or infected soil). PLS-DA is a modified version of PLSR and is mainly used for classification purposes. Here, we used PLS-DA to develop a classification model for the control and infected groups.

#### 2.4.2 Selection of main wavebands for classification

The variable importance in projection (VIP) and successive projection algorithm (SPA) methods were applied to extract the main wavebands related to root rot infection. We applied the combined VIP and SPA methods as an ensemble filtering algorithm. The VIP represents the contribution of each waveband—as determined by the optimal LVs in the PLS-DA—as a VIP score. Wavebands with a VIP score ≥1 were determined to be the main wavebands (Chong and Jun, 2005). The SPA was obtained in the PLS-DA-based model, in which the main wavebands were determined based on the VIP score. The SPA algorithm prevents collinearity and extracts the main wavebands with minimum overlap in a multiple linear regression model (such as PLS-DA) using reduced data resources (Araújo et al., 2001; Soares et al., 2013).

### 2.5 Image processing

#### 2.5.1 Data classification

A PLS model based on averaged spectral data is often not applicable to the HSI threshold. This happens when there is a large deviation between the average values and the original HSI data, when there is some level of noise in the model (owing to a weak spectral signal), or when there is a large deviation in the weighted values in the PLS-DA model. To resolve this, we first applied the PLS-DA model to the original HSI data and investigated the resultant values which are distributed in the rage from -0.5 to 1.5 based on the frequency distribution. The distribution of the entire classification values had a shape similar to that of a normal distribution. Therefore, data in the region encompassing ±2σ (approximately 95.5%, based on the median) were used in subsequent analysis, and data in the remaining region were considered noise.

#### 2.5.2 Image enhancement

When the sensor emits a weak signal for a specific wavelength or when the measured intensity is weak, the HSI data may contain latent (invisible) noise that can be visualized and removed. During image registration using the PLS-DA model, the image for each wavelength was multiplied by the weights of the model, following which all images of all wavelengths were added (Mo et al., 2014). At this time, the latent noise in the image was visualized. Most of the noise appeared in a linear shape (similar to a notch). In addition, most of these data points were outliers (very low or very high values) that were unrelated to the target object and had a negative effect on identifying patterns in the image. To resolve this, the images were improved through selective filtering using a notch rejection filter to remove noise generated in the SWIR region (Aizenberg and Butakoff, 2008; Park et al., 2019).

## 3 Results

### 3.1 Root rot infection and pathogen activity

Pathogen activity was investigated to determine whether plants in the control and infected groups were infected with *Cylindrocarpon destructans*. Pathogen density was evaluated based on gene expression levels using real-time PCR. Samples of infected and sterilized soil and of ginseng root samples from plants grown in each soil type were used for real-time PCR analysis (**Table 1**). The number of gene copies (SQ, representing pathogen density) was significantly different between the control and infected groups. This indicated a difference in pathogen activity and confirmed that the pathogenic treatment was successful. According to the Rural Development Administration of Korea, the possibility of root rot infection is very high when the SQ value of the soil is >100 Lee S. H. (2021). Our results confirmed that the experimental treatment was effective according to this threshold.

**Table 1:** SQ values in ginseng roots and soil samples infected with *Cylindrocarpon destructans*.

	Soil		Root	
	Control	Infected	Control	Infected
SQ	1.80 ± 1.35	115.56 ± 61.00	0.10 ± 0.00	14.07 ± 9.54

SQ: starting quantity (number of gene copies) in soil or root samples (1 g).

Controls are intact roots and soil samples without the pathogen.

**Figure 1:**
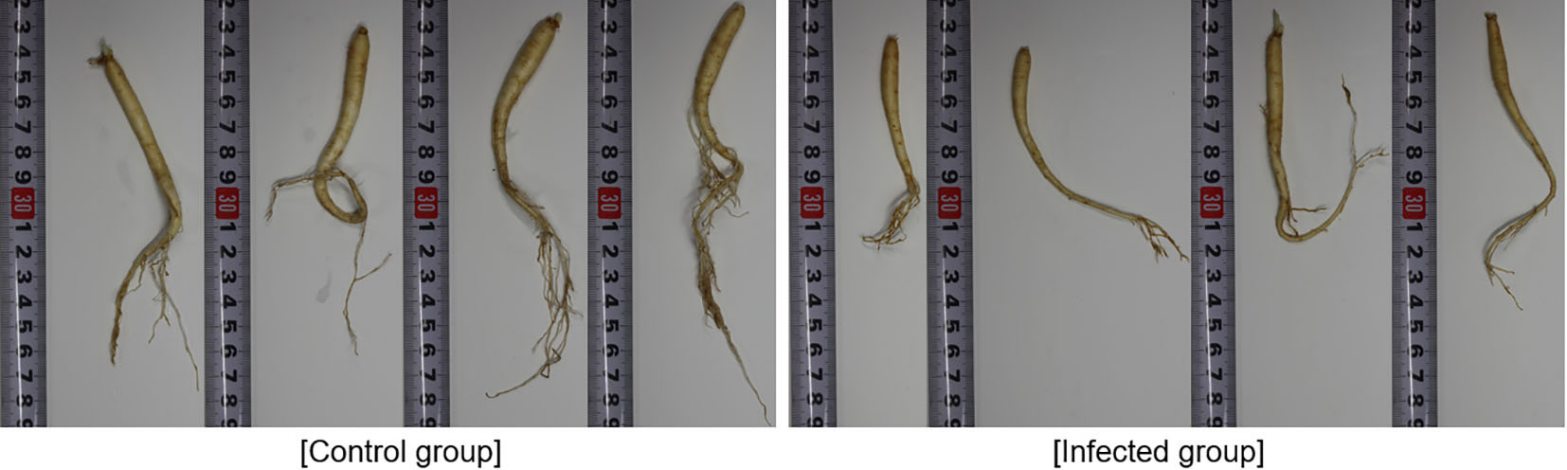
Comparison of root development in control and infected ginseng plants (unit: cm).

The actual state of the root is shown in **Figure 1**. In the control group, the average length of the major axis of the roots was approximately 15 cm (**Figure 1A**), and the plants showed good fine root development. However, in the infected group, the average length of the major axis was approximately 10 cm (**Figure 1B**), and the plants showed poor fine root development. Therefore, it was possible to confirm the status of root development based on the treatment applied to infected soil.

### 3.2 Spectral profile of ginseng leaves

**Figure 2:**
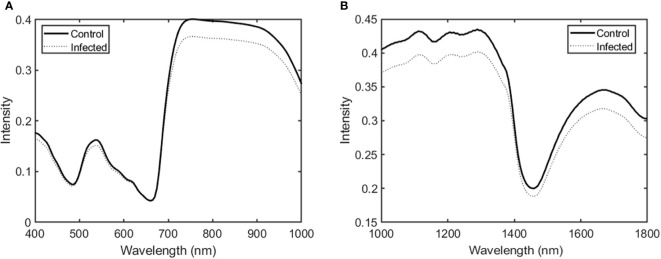
Average spectra in the **(A)** Vis/NIR and **(B)** SWIR regions for ginseng leaves in the control and infected groups.

**Figure 2** shows the averaged spectral data in the Vis/NIR and SWIR regions for ginseng leaves in the control and infected groups. Overall, spectral intensity in the Vis/NIR and SWIR regions was higher in the control group than in the infected group. Significant differences appeared in the 700–900 nm range (Vis/NIR region) and in the 1000–1300 and 1600–1700 nm ranges (SWIR region).

### 3.3 Results of the PLS-DA model and coefficients of the main wavebands

#### 3.3.1 Vis/NIR data analysis

**Figure 3:**
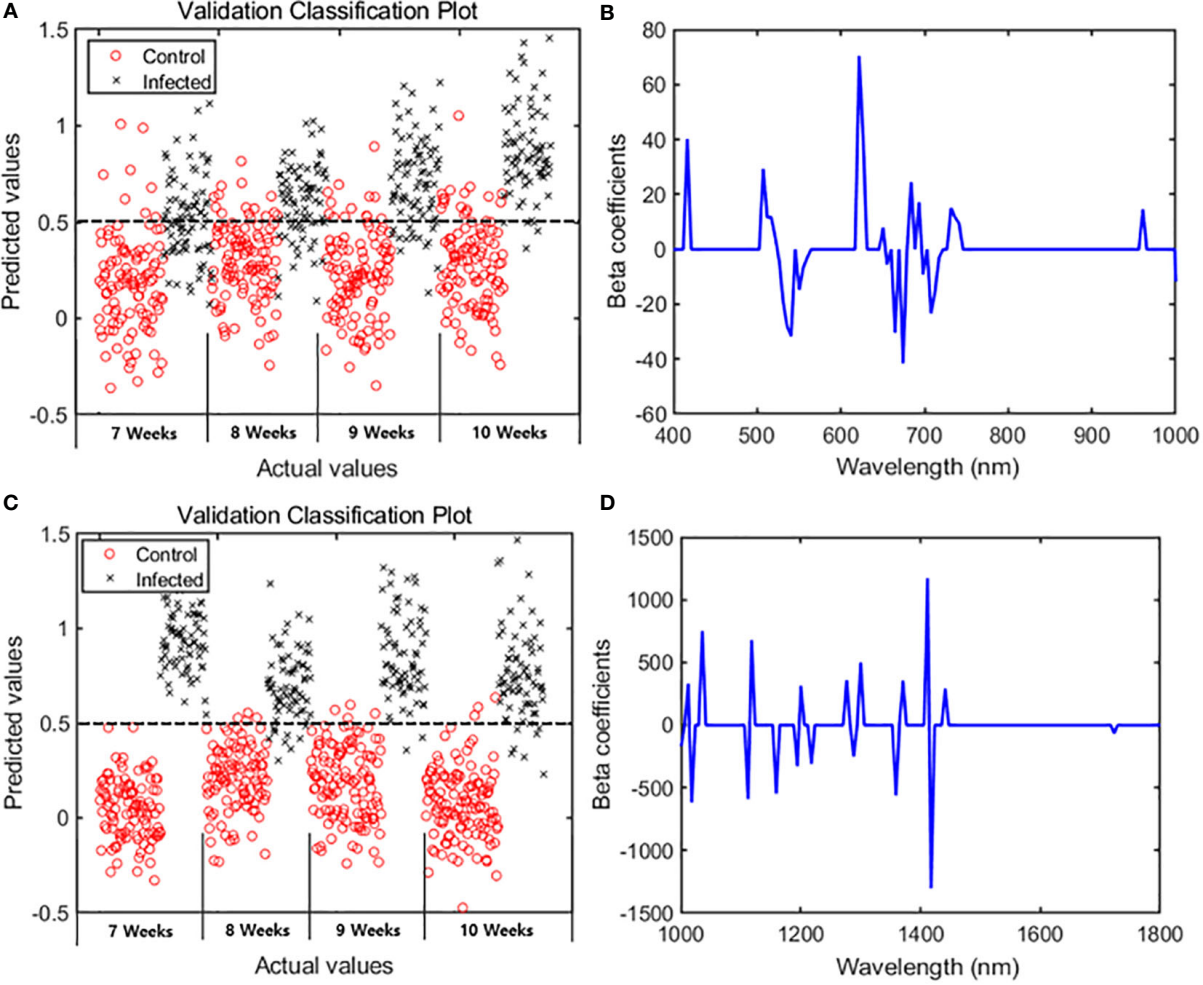
PLS-DA results showing 29 spectral bands in the Vis/NIR region and 19 spectral bands in the SWIR region. Classification plots for validation in the **(A)** Vis/NIR and **(C)** SWIR regions. Beta coefficients from the PLS-DA results in the **(B)** Vis/NIR and **(D)** SWIR regions.

The main wavelengths were extracted by applying the VIP–SPA filtering to PLS-DA, and the accuracy was summarized for total and time series data (**Table 2**). The performance was evaluated based on the classification model developed with the spectral data of the entire period. In the VIP–SPA analysis, the Vis/NIR region showed a total accuracy of 81.1% (**Table 2a**). In the weekly time series analysis (**Table 2b** and **Figure 3A**), the Vis/NIR region showed a discrimination accuracy of 76%, 80%, 85%, and 87% at weeks 7–10, respectively. These results indicated a pattern of increasing accuracy over time. The weighted area of the main wavelength included the following 29 wavelengths (**Figure 3B**): 417, 502, 512, 517, 522, 526, 531, 536, 541, 550, 555, 560, 622, 627, 657, 650, 665, 674, 684, 693, 698, 708, 713, 717, 732, 736, 741, 961, and 1004 nm.

**Table 2:** Main wavebands in the Vis/NIR and SWIR regions, as identified by the VIP–SPA analysis of (a) all time and (b) weekly time series analysis.

			Total			Correct		Accuracy (%)		
			All	Con**	Inf**	Con	Inf	Con	Inf	Overall
(a)	Vis/NIR	Cal*	1471	843	628	730	475	86.6	75.6	81.1
		Val*	750	429	321	374	242	87.2	75.4	81.3
	SWIR	Cal	1467	879	558	867	563	98.6	95.7	97.2
		Val	750	447	303	436	282	97.5	93.1	95.3
(b)	Vis/NIR	7 Weeks	188	107	81	99	44	92.5	54.3	76.1
		8 Weeks	189	109	80	94	58	86.2	72.5	80.4
		9 Weeks	190	108	82	95	67	88.0	81.7	85.3
		10 Weeks	183	105	78	86	73	81.9	93.6	86.9
	SWIR	7 Weeks	180	103	77	103	77	100.0	100.0	100.0
		8 Weeks	177	103	74	99	64	96.1	86.5	92.1
		9 Weeks	194	119	75	115	73	96.6	94.3	96.9
		10 Weeks	199	122	77	119	68	97.5	88.3	94.0

*Calibration and validation.

**Control and infected groups.

#### 3.3.2 SWIR data analysis

In the VIP–SPA analysis, the SWIR region showed a total accuracy of 95.3% (**Table 2a**). In the weekly time series analysis (**Table 2b** and **Figure 3C**), the SWIR regions showed discrimination accuracies of 99%, 92%, 97%, and 94% at weeks 7–10, respectively. These results indicated that the SWIR region showed better predictive performance than the Vis/NIR region, with >90% accuracy across weeks. The weighted area of the main wavelength included 19 wavelengths (**Figure 3D**): 1001, 1012, 1018, 1036, 1112, 1118, 1159, 1194, 1200, 1217, 1277, 1288, 1300, 1359, 1371, 1412, 1417, 1441, and 1723 nm.

#### 3.3.3 PLS-DA-based image analysis for detecting fungal infection

**Figure 4:**
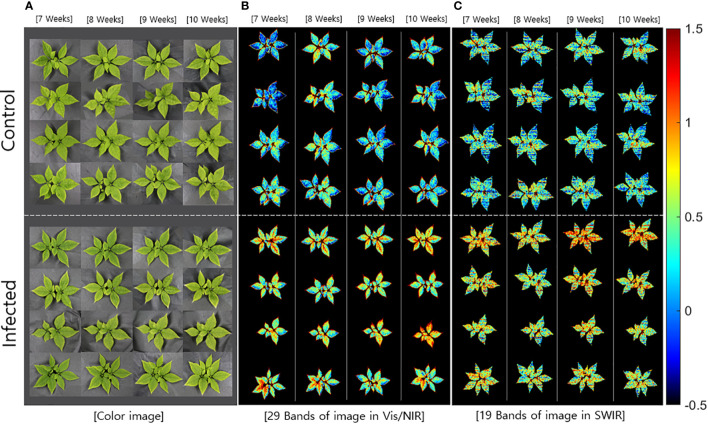
Comparison of **(A)** color images and multispectral images with data from the main wavebands in the **(B)** Vis/NIR (29 bands) and **(C)** SWIR regions (19 bands).

The beta coefficients obtained from the PLS-DA (**Figure 3**) were applied to the hyperspectral image, which was subsequently expressed as a multispectral image (**Figure 4**). The color images (**Figure 4A**) were significantly different from the multispectral images that included data from 29 wavelength combinations in the Vis/NIR region (**Figure 4B**), thus confirming differences in HSI data between the control and infected ginseng plants. The control group did not show any major changes across weeks, whereas the infected group changed to a red color, indicating gradual damage due to fungal infection (**Figure 4B**). These changes were most marked at the tip and center of each leaf. The multispectral images in the SWIR region (including 19 wavelength combinations; **Figure 4C**) showed some noise. Nevertheless, there was a clear difference in color between the control and infected groups.

#### 3.3.4 Biochemical interpretation of selected wavelengths

Among the main wavelengths in Vis/NIR region, the 417 nm band is similar to that associated with chlorophyll a content (Kawamura et al., 2008), and the 512–560 nm bend reflects overall chlorophyll content. Additionally, 541 and 560 nm are related to anthocyanin absorbance (Ndisya et al., 2021). The 622–698 nm band is known as the red region, and the 665, 684, and 708 nm wavelengths are similar to those associated with chlorophyll a content (Thenkabail et al., 2000). The 708–741 nm band corresponds to the red edge region, and the 713 nm band is highly correlated with protein and nitrogen levels. In addition, the 736 nm wavelength is associated with OH, and 741 nm is related to CH reactions (Shenk, 1992). The 961 nm wavelength associated with an OH reaction that is close to the region related to water and starch reactions (970 nm) (Kawamura et al., 2008). Moreover, 512, 541, 560, 693, 713, and 741 nm are major wavelengths that can be used to predict the total chlorophyll content (Wang et al., 2019b).

The SWIR region contains different wavelengths related to the organic components of plant leaves. In this study, the 1001 nm region was related to OH, whereas the 1012, 1018, and 1036 nm regions were related to the CH functional group. The 1018 nm region is known to be related to proteins, and the 1036 nm region is known to be related to oils (Shenk, 1992). The 1112–1300 nm region reflects a combination of the CH and OH functional groups and is also related to the chemical structure of phenolic compounds in crops (Dale et al., 2013). As such, changes in phenolic compounds associated with wavelengths in this region may indicate symptoms of infection. The 1200 nm wavelength is related to water, cellulose, starch, and lignin, whereas 1217 nm is related to starch (Kawamura et al., 2008). The 1359 nm region is related to CH, 1412 nm to OH, 1441 nm to sucrose and starch, and 1723 nm to the CH functional group (similar to that of carotenoids) (Xiaobo et al., 2010) and chlorophyll (Ji-Yong et al., 2012).

## 4 Discussion

Generally, exposure to abiotic and biotic stresses can induce various changes in plants, including in factors such as photosynthetic activity and the levels of carotenoids, anthocyanins, moisture, and starch. These changes can be detected optically through the analysis of spectral bands either in the Vis-NIR or SWIR region.

The main wavelength of Vis/NIR region associated with the chlorophyll in this study was similar to those associated with high-temperature stress in ginseng (Park et al., 2021). However, the main wavelengths related to anthocyanins (541 and 560 nm), nitrogen and protein (708–740 nm), and moisture and starch (961 nm) were different from those reported previously. These results can be confirmed by comparing the specific wavelengths related to root rot with those related to high-temperature stress in the Vis/NIR region. In the SWIR region of carotenoids, chlorophyll, protein, and water wavebands were confirmed similar to those observed in the Vis/NIR region. Similarly, the wavelengths related to starch (1217 and 1441 nm) were the same as those related to high-temperature stress. However, there were differences in the 1112–1300 nm range (related to phenolic compounds) and 1723 nm (related to carotenoids and chlorophyll).

The results indicated that when ginseng is infected by root rot, the negative effects on root development cause a decline in nutrient absorption. It may lead to a decrease in overall photosynthetic activity and promotes carotenoid expression. Differences in the main components of primary and secondary metabolites—such as starch, sucrose, glucose, and amino acids—are also expected. In addition, the increase of phenolic compounds can be observed, which are synthesized as a result of plant resistance to pathogen infection and disease.

In most of the cases, nutrient absorption and growth activity decrease when plant roots are infected, and the onset of resistance disease. The spectral patterns of ginseng leaves infected by root rot disease could be influenced by physiological changes of the photosynthetic activity and the levels of carotenoids, anthocyanins, moisture, starch, and phenolic compounds. The changes could be investigated through spectral analyses in this study.

Hyperspectral imaging technique has an advantage of being able to detect and show biochemical changes in organisms more sensitively than conventional color imaging. However, the relative high cost of the hardware and the large image data to be processed have been a hamper for HSI technique to be used in agricultural industry widely.

## 5 Conclusions

In this study, we used HSI data in the Vis/NIR and SWIR regions (400–1800 nm) to develop a model for the early prediction of root rot disease in ginseng. The main wavelengths were selected based on PLS-DA supplemented by VIP–SPA analysis. In the Vis/NIR region, the average accuracy of the model (using 29 wavelengths) was 84%, and the accuracy increased every week over a period of four weeks. In the SWIR region, the average accuracy of the model (using 19 wavelengths) was 95%, although we did not observe any special patterns in these results.

The use of HSI technology allows us to observe the spectral changes in a non-destructive manner in ginseng plant induced by root rot disease. Further chemical analyses need to be performed for the verification of the changes in biochemical content of the infected ginseng plant. We expect that the techniques proposed in this study can be used for the early prediction of root rot and other soil-borne diseases in ginseng plants.

## Data availability statement

The raw data supporting the conclusions of this article will be made available by the authors, without undue reservation.

## Author contributions

EP and B-KC conceived the overall contents and structure for this article. EP and Y-SK led the data analysis drafted tables and figures. MF, MK, and IB analyzed sample information and conducted experiment. EP and B-KC reviewed successive drafts. All authors contributed to the article and approved the submitted version.
